# The systematic error caused by random errors through data reduction

**DOI:** 10.1155/S1463924687000051

**Published:** 1987

**Authors:** K. M. Hangos, L. Leisztner

**Affiliations:** 1 Computer and Automation Institute Hung. Acad. Sci. P.O. Box 63 Budapest H-1502 Hungary; 2 Institute of Forensic Sciences P.O. Box 314/4 Budapest H-1903 Hungary


Journal of Automatic Chemistry/*Journal of Clinical Laboratory Automation*, Vol. 9, No. 1 (January–March 1987), pp. 25–29

### The systematic error caused by random errors through data reduction

**K. M. Hangos**

*Computer and Automation Institute, Hung. Acad. Sci., H-1502 Budapest, P.O. Box 63, Hungary*

**and L. Leisztner**

*Institute of Forensic Sciences, H-1903 Budapest, P.O. Box 314/4, Hungary*

### Introduction

During the evaluation of the continuous measurement signal of analytical instruments by a digital computer, the signal is sampled periodically, and the analytical information [1] is computed from this sequence of discrete values representing the signal by the procedure called data reduction [2]. For example, retention data, peak heights and/or peak areas are produced from the raw discretized signal, in the case of chromatogram evaluation.

Of course the analytical information produced should not contain a systematic error component, that is, replicate measurements should give more precise information. Measurement conditions are chosen so that the raw signal of the measurement device, as represented by the sequence of discrete values, does not contain a systematic error component. As measurement is a complex process, its component processes can add random errors with various distributions. These random errors can be transformed into systematic error components [3]. The random error of the raw signal can also be transformed into a systematic error component in the analytical information by the data processing: Enke and Nieman [4] investigated this effect in the case of data smoothing, and Eisenhart [5], Ivanova and Tkatchev [6] studied it in the case of calibration. This paper reports an investigation of data reduction transformation causing a systematic error component from random errors. The work used mathematical and simulation methods.

### Mathematical modelling of data reduction

The aim of data reduction is to compress the raw data produced by the measurement device and to bring it to a form suitable for evaluation without significant loss of the analytically important information. A loss of information is unavoidable during the analytical information production step of data reduction. This is because not all of the information contained in the measured raw data is necessary – the information content of the analytical information is orders of magnitude smaller.

As a measurement data model a so-called discrete time vector valued stochastic process (stochastic sequence) was chosen, because the raw continuous time measurement signal is usually sampled as the first step of evaluation by digital computers. In order to handle further formulae more simply let us assume that the discretized signal does not contain outliers and systematic error components, also let us investigate the one-dimensional case. (Extension to the multivariate case does not cause any difficulty.) The model of the measurement signal can thus be written in the form:

$$x(t) = X(t) + s.e(t) \quad t = 1,2,....,T \tag{1}$$

where: $x(t)$ is the stochastic sequence of the discrete measurement signal
$X(t)$ is the true value sequence
$e(t)$ is the white noise stochastic sequence with standard normal distribution (with independent elements, 0 mean and 1 variance)
$s^2$ variance of the random measurement error
$T$ number of samples.

Our assumption about the absence of outliers and systematic error components does not limit the generality of our conclusions. If it can be shown that in this simple (special) case the data reduction does transform the random error contained in the measurement signal, into systematic error in the analytical information, then in the more complicated case the analytical information will at least contain this sytematic error component.

During the data reduction step a new stochastic sequence is obtained from the measurement sequence (1), by a transformation determined by the type of data reduction chosen. This sequence (2) contains the analytical information:

$$y(k) = R\{k; x(1), x(2),...,x(T)\}, k = 1,...K \tag{2}$$

where $K \ll T$. The transformation $R\{.;.,.,...,.\}$ is called the data reduction transformation.

The data reduction transformation is in the general case a nonlinear one. According to the well-known definition of linear transformation, a data reduction transformation is linear if multiplication of the sequence by a constant before or after transformation gives the same result and the sum of two transformed sequences is equal to the transform of the sum of the two sequences.

The true value sequence of the analytical information sequence $\{y(k)\}_{k=1}^{K}$ can be defined as if its element would be obtained from the true value of the measurement sequence by the data reduction transformation (2).

As the data reduction transformation is nonlinear in the general case, the mean value of the analytical information sequence is not equal to its true value, so:

$$E[y(k)] = E[R\{k;x(1),..,x(T)\}] \# \\ \# R\}k;E[x(1)],..,E[x(T)]\{ = R\}k;X(1),..,X(T)\{ = Y(k) \quad (4)$$

Thus in the case of nonlinear data reduction transformations the random error component in the measurement sequence causes a systematic error component in the analytical information sequence. This systematic error component can be theoretically eliminated by a suitably chosen correction, but the problem is not solved for data reduction transformations used in analytical chemical practice.

At the same time, in the case of linear data reduction transformations, the random error in the measurement sequence does not cause systematic error in the analytical information. In this case the data reduction transformation has the following simple form:

$$y = \underline{\underline{R}}(t) \,.\, x \quad (5)$$

where $y$ and $x$ denote the vectors composed of the elements of the analytical information and measurement sequence respectively, $\underline{\underline{R}}(t)$ is the matrix of the data reduction transformation with only time dependent elements. Repeating the derivation of Equation (4) for this case, using the fact that the elements of the random error sequence are independent, we obtain:

$$E[y] = E[\underline{\underline{R}}(t).x] = \underline{\underline{R}}(t).E[x] = \underline{\underline{R}}(t).X = Y \quad (6)$$

In this case there is no systematic error in the analytical information sequence.

### Data reduction transformations for the reduction of discretized chromatographic raw data

In the discussion of the data reduction of measurement sequences obtained from chromatographs, the components of the measurement sequence have to be taken into account. For the sake of simplicity let us assume that the measurement sequence contains only the signal of the unknown compounds as peaks with the shape of Gaussian distribution function, an additive random error and a base-line component:

$$x(t) = \sum_{j=1}^{J} (q(j).f[(t - u(j))/z(j)]) + c(t) + s.e(t);\ t = 1,...,T \quad (7)$$

where: $u(j)$, $z(j)$ are parameters characterizing the quality of the compounds

$q(j)$ is the parameter characterizing the amount of the compounds

$c(t)$ is the base-line sequence (deterministic).

As analytical information for the determination of the amount of the $j$th compound, the peak height:

$$H(j] = X\,(u(j)) - c(u(j)) = q(j] - c(u(j)),$$

or the peak area is used. For the determination of the influence of the data reduction process, it can be divided into well-defined subprocesses (elementary transformations), which are:

(1) Peak recognition.

(2) Base-line correction.

(3) Calculation of peak height or area.

The data reduction transformation can be regarded as a composite of the transformations listed above in the given order. Let us first investigate the elementary transformations of the composite data reduction transformation separately.

The peak recognition algorithm is used for the decision whether or not a peak is beginning or ending at a given time $t$, as parameters $u(j)$, $j = 1,...,J$ are unknown. The peak recognition algorithm separates a set of signal samples belonging to the peak, and the maximum or the integral area is computed. For this purpose a moving group of consecutive points from the $j$th to the $s$th sample are separated as being before and after peak (typically up to 30 sample values), their mean is calculated $\bar{x}(t_j - t_s,t_j)$. A start or end of peak is assumed if the deviation of two consecutive samples exceeds a given limit $m$, from the mean value:

$$|\, x(t_j + k) - \bar{x}(t_j - t_s,t_j)\,| > m \quad k = 1,2 \quad (8)$$

Furthermore the peak is accepted if it has a maximum and if its area exceeds a specified minimum value. It must be noted here that other peak detection algorithms (i.e. based on the value of the derivatives of the sequence) can also be used.

The difference of consecutive signal samples depends not only on the deterministic component ($X(t_j + 1)$ and $X(t_j + 2)$), but also on the random error ($e(t_j + 1)$, $e(t_j + 2)$) and the base-line value. This means that the number of samples assumed to belong to the peak is a random variable, which will be denoted by $n(t)$. It is easily seen, that $n(j)$ is a nonlinear function of the measurement signal samples, because in the case of twice as large $q)j)$, the number of samples belonging to the peak will not be twice larger because of the nonlinear (Gaussian) shape of the peak. Thus this peak recognition step is a source of systematic error.

The data in table 1 show the variation of the number of samples assumed by the given algorithm to belong to the peak in simulated chromatograms as a function of the various parameters of the peak. (The description of the simulation experiment is given in the next section.) Data in the column designated H/W = 1000 refer to 'narrow' peaks. It can be seen that the variation of the number of samples included does not vary very much, but also that the variation is not a monotonous function of signal-to-noise ratio (it first increases then decreases with increasing signal to noise ratio). At the same time the uncertainty of peak recognition decreases with increasing signal-to-noise ratio in every column as demonstrated by the value of the standard deviation $S$. For wide peaks the number of samples assumed to belong to the peak increases very rapidly with increasing signal-to-noise ratio. This property becomes stronger as the peak width increases.

For the computation of the peak height, as well as the peak area, the value of the base-line must be determined. As a peak covers up the base-line a base-line correction algorithm is used for estimating the values of the base-line, based on the samples before and after the peak. In the most simple (linear) case, the algorithm selects two groups of samples from the measurement sequence of predetermined size, before and after the peak, computes the means of the groups, orders these averages to the centre of the groups in the $t$-domain as points of the base-line, and connects the two with a straight line which will serve as the estimate of the base-line between the two points. The above base-line correction algorithm is a linear transformation of the selected samples in the two groups into the parameters of the line (the computation only requires addition, subtraction and multiplication by constant values). Also the substitution into the linear base-line requires only linear data manipulation. At the same time, there are well known nonlinear base-line correction algorithms (i.e. the algorithm based on polynomial fitting), in which cases both the computation of the base-line and the substitution into the nonlinear base-line are nonlinear transformations.

It is important to note, that the systematic error of the peak recognition affects the base-line correction algorithm, because the groups before and after the peak should be as close as possible to the peak. So samples belonging to the peak can be assumed to be in one of the surrounding groups.

As the final step, the calculation of the peak area from the $n(j)$ selected samples is a linear transformation if a constant or linear form of base-line is assumed:

$$A(j) = h. \sum_{k=tj}^{tj+n(j)} (x(k) - c(k)) \quad (9)$$

In this case there is no further nonlinear transformation causing possible systematic error in this step.

the calculation of the peak height from the selected $n(j)$ samples is a nonlinear data transformation as the determination of the peak height is usually computed by fitting a parabolic curve to a set number of the samples having the largest values, the peak height being taken to be equal to the height of the parabola. The above algorithm filters the effect of the random error component in the case of Gaussian distributed random errors according to the least squares estimation properties (for other distributions it is only approximately valid), but the estimation of the parameters of the parabola from the measurement samples is a nonlinear transformation because of the nonlinear character of the parabola. For this reason the estimation of the peak height is also a nonlinear data transformation, so it can introduce systematic error components from the random errors.

At the same time the systematic errors of the base-line correction algorithm appears in the value of the peak height calculated as above, transferring the systematic error caused by the peak recognition step. However, the systematic error in the estimated base-line affects the peak height calculation much less than it does the peak area, as in the latter case the number as well as the values of the measurement signal samples belonging to the peak distort the result of the peak area calculation.

The above explains the well-known empirical fact that only the peak height, and not the peak area, can be used for quantitative trace analysis (Hachenberg [7]). In trace analysis the signal-to-noise ratio is rather small.

### The effect of data reduction on the result of chromatographic analysis as a function of the parameters of the chromatogram

The effect of data reduction was investigated by computer simulation of measurement signal sequences with known variances. The simulations were performed on a HP 21MX computer.

It was assumed for the computations that the function describing peak shape is a Gaussian distribution function. In order to model the qualitative and quantitative deviations of the compounds, several Gaussian distribution functions were used, which can be characterized by their height $H$ and the interval $W$ cut from the base-line by the tangents at the inflexion points. The distribution of the random error component was chosen to be Gaussian and uniform. As the results for the two cases coincide, only the results of the case with Gaussian functions are shown here. The other parameters of the simulation experiment are detailed elsewhere [8 and 9].

The limits for the parameters investigated were the following:

$H/W$: 10–1000 mV/s
$W$ : 4–32 s
$E$ : 10–$\infty$

The simulation results indicate that the systematic error caused by the random error component in the measurement signal samples generally increase with the decrease of the signal-to-noise ratio $E$, which in our case can be defined after Smit [10] as follows:

$$E = H/s \quad (11)$$

At the same time magnitude and sign of the systematic error varies as a function of peak shape, i.e. as a function of the height/width ratio ($H/W$).

Figures 1–3 show the variation of the relative peak height ($\Delta H/H$) and relative peak area ($\Delta A/A$) as a function of the signal-to-noise ratio for peaks with different shapes.

Figure 1 contains data for narrow peaks (high $H/W$), for which there are relatively low numbers of signal samples belonging to the peak, having a relatively rapid deterministic change. It can be seen that both the relative peak height and area increases with decreasing signal-to-noise ratio. It should also be noted from the data in table 1, that there is only a relatively small variation in the number of samples assumed to belong to the peak, especially when relative values are examined.

It can be seen from figure 2 that in the case of medium wide peaks (intermediate values of $H/W$), the peak height increases with decreasing signal-to-noise ratio, while the peak area decreases after a short period of increase.

In the case of broad peaks (low values of $H/W$) the number of samples belonging to the peak is large. The data in table 1 show that in this case the number of samples assumed to belong to the peak reduces very rapidly with decreasing signal-to-noise ratio. So for broad peaks both the peak height and area reduce with decreasing signal-to-noise ratio.

### Discussion

It can be seen from the simulation results that the signal-to-noise ratio required for a negligible bias to be introduced by the transformation of the random error of

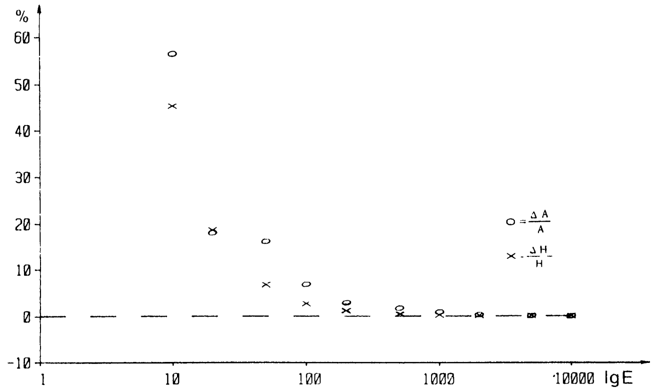

*Figure 1. The effect of the signal-to-noise ratio (E) on the peak height and area (H/W = 1000 mV/s, with Gaussian distributed random error, each point representing the mean of 10 simulated values). Where H = peak height; ΔH = variation of the peak height compared to the theoretical (true) value; A = peak area; ΔA = variation of the peak area compared to the theoretical (true) value; W = peak width.*

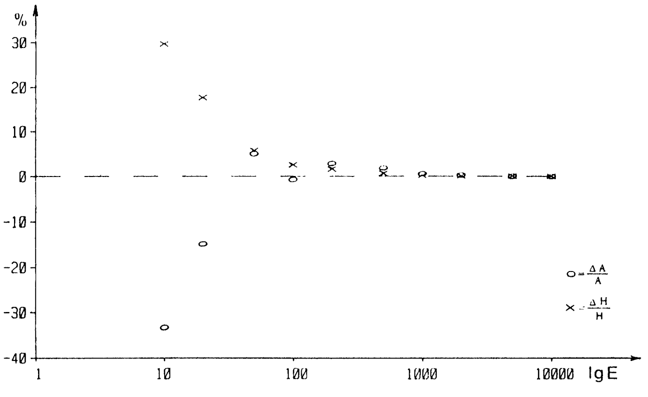

*Figure 2. The effect of the signal-to-noise ratio (E) on the peak height and area (H/W = 600 mV/s). Other details same as in figure 1.*

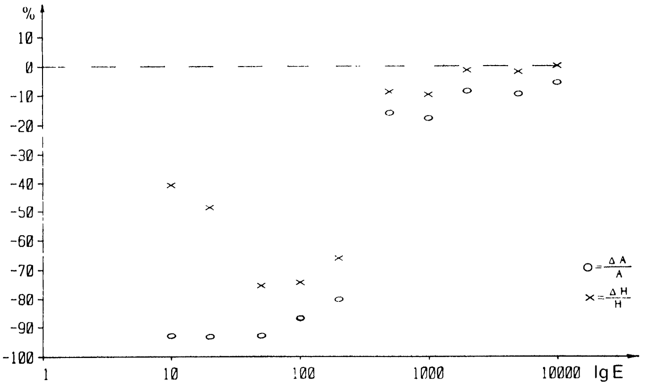

*Figure 3. The effect of the signal-to-noise ratio (E) on the peak height and area, (H/W = 60 mV/s). Other details same as in figure 1.*

the measurement signal samples varies with the chromatographic peak shape. For example the signal-to-noise ratio normally achievable in analytical practice (around 500) is sufficient in the case of narrow peaks, but it should be greater than several thousands for wide peaks. In the case of chromatographic peak height evaluation, these

*Table 1. The number of measurement signal samples (N) assumed by the integration algorithm to belong to the peak as a function of the signal-to-noise ratio (E). Each point represents the mean of 10 simulated values. Where S (%) = the relative standard deviation; H = the peak height; W = the peak width.*

| *H/W* mV/s | 1000 | | 600 | | 240 | | 60 | |
|---|---|---|---|---|---|---|---|---|
| *E* | *N* | *S%* | *N* | *S%* | *N* | *S%* | *N* | *S%* |
| 10 | 41,8 | 40,4 | 27,9 | 31,4 | 22,6 | 20,3 | 30,2 | 32,3 |
| 20 | 37,0 | 32,5 | 50,6 | 42,5 | 37,7 | 53,4 | 31,3 | 46,4 |
| 50 | 55,6 | 10,5 | 77,6 | 10,3 | 88,4 | 56,1 | 57,2 | 58,4 |
| 100 | 60,9 | 8,3 | 93,2 | 16,2 | 162,6 | 19,2 | 102,8 | 55,0 |
| 200 | 67,0 | 20,7 | 118,5 | 11,9 | 200,5 | 26,5 | 227,0 | 26,0 |
| 500 | 73,4 | 19,4 | 160,7 | 14,0 | 234,8 | 7,4 | 318,6 | 16,0 |
| 1000 | 89,6 | 19,2 | 137,4 | 5,3 | 231,0 | 3,8 | 316,2 | 13,6 |
| 2000 | 82,4 | 11,6 | 147,4 | 8,9 | 236,4 | 1,8 | 332,6 | 4,5 |
| 5000 | 78,8 | 10,4 | 129,5 | 1,6 | 239,9 | 0,7 | 326,9 | 3,7 |
| ∞ | 69,0 | 0,0 | 133,0 | 0,0 | 242,0 | 0,0 | 350,0 | 0,0 |

values can be slightly lower. For comparison purposes it should be noted that the best signal-to-noise ratio achievable in spectrodensitometry of thin layer chromatograms is not greater than 500, because of the significant noise of optical origin [11]. At the same time, the signal-to-noise ratio of the main components in gas and modern liquid chromatography can reach the range of a couple of ten thousands, with suitable detector sensitivities and retention times. In the case of chromatographic trace analysis, the signal-to-noise ratio is usually less than 50. For details of practical applications see Leisztner *et al.* [9].

### References

1. Veress, G. E., Bezegh, A. and Pungor, E., Analytical chemical measuring systems. In *Progress in Cybernetics and Systems Research, Vol. XI* (Hemisphere Publishing Corporation, Washington, 1982).
2. Perone, S. and Jones, D. O., *Digital Computers in Scientific Instrumentation, Applications to Chemistry* (McGraw-Hill Book Company, New York).
3. Kolthoff, I. M. and Elving, P. J. (Eds), *Treatise on Analytical Chemistry, Part I* (John Wiley & Sons, New York–Chichester–Brisbane–Toronto, 1978).
4. Enke, C. G. and Nieman, T. A., *Analytical Chemistry*, **48** (1976), 704A–712A.
5. Eisenhart, C., *Ann. Math. Statist.*, **10** (1939), 162–186.
6. Ivanova, T. I. and Tkatchev, Y. A., Trudy Institute Geology y Geophysicy Acad. Sci. SSSR, Syb. Otdiel. No. **450** (1981), 55–59.
7. Hachenberg, H., *Industrial Gas Chromatographic Trace Analysis* (Heyden, London, 1973).
8. Leisztner, L., Barna, P. and Ullrich, E. *Journal of HRC and CC*, **5** (1982), 379–380.
9. Leisztner, L., Kuzmin, N. M. and Barna, P., *Zhurnal Analiticheskoj Himii*, **38** (1983), 2247–2256.
10. Smit, H. C. and Walg, H. L., *Chromatographia*, **8** (1975), 311–323.
11. Leisztner, L., Kuzmin, N. M. and Szalma, E., *Zhurnal Analiticheskoj Himii*, **37** (1982), 1384–1392.

**38th Pittsburgh Conference and Exposition**
*9–13th March 1987: Atlantic City, USA*
The agenda of sessions for the 1987 Pittcon includes:

**March 9**
SYMPOSIUM – Computer-Aided Microscopy and Analysis
SYMPOSIUM – Occupational Health and Safety in the Laboratory
SYMPOSIUM – Reflectance Infrared Spectroscopy
SYMPOSIUM – Instrumentation and Automation of Environmental Sample Analysis
SYMPOSIUM – Multidimensional Separations
SYMPOSIUM – Nuclear Magnetic Resonance in Solids

**March 10**
SYMPOSIUM – The Analytical Chemistry Opportunity in Process Instrumentation
SYMPOSIUM – ASTM E-42 – Hybrid Analytical Techniques Involving Surface Analysis
SYMPOSIUM – Pittsburgh Analytical Chemistry Award – Future Directions in Mass Spectrometry
SYMPOSIUM – Chemometrics in the Computer-Integrated Laboratory
SYMPOSIUM – 1987 Dal Nogare Award
SYMPOSIUM – Williams-Wright Award

**March 11**
SYMPOSIUM – New Developments in Fourier Transform Mass Spectrometry
SYMPOSIUM – Pittsburgh Spectroscopy Award and Maurice F. Hasler Award
SYMPOSIUM – Stationary Phase Structure and Retention in Reversed Phase Liquid Chromatography
SYMPOSIUM – LC/MS and SFC/MS – Will They Replace GC/MS?
SYMPOSIUM – Charles N. Reilley Award
SYMPOSIUM – Separation Science and Technology: Metals

**March 12**
SYMPOSIUM – Advances in Raman Spectroscopy
SYMPOSIUM – Detectors for LC and SFC
SYMPOSIUM – Symposium Honoring the Late Tomas Hirschfeld
SYMPOSIUM – Inductively Coupled Plasma-Mass Spectrometry
SYMPOSIUM – Immobilized Reagents in Chemical Analysis
SYMPOSIUM – Quality and Productivity in the Analytical Laboratory

**March 13**
SYMPOSIA – User-Manufacturer Information Exchange
Advances in Supercritical Fluid Chromatography
Applications of Commercial Computer Systems
Computers and Laboratory Management
General Analysis
HPLC-Dectection and Optimization
HPLC-Hardware
MS-MS and SIMS
New Advances in GC Instrumentation and Automation
Raman Spectrsocopy II
Robotics-Applications and System Updates
Trace Analysis-Biological and Environmental

*Details from Pittsburgh Conference, Dept. UPD, 12 Federal Drive, Suite 322, Pittsburgh, PA 15235, USA. Tel.: 412 795 7110.*

